# Spatial Transcriptomics for Dissecting Cellular and Molecular Heterogeneity in the Aging and Diseased Brain

**DOI:** 10.3390/ijms27146149

**Published:** 2026-07-09

**Authors:** Seeun Cha, Jin Kim, Jisan Kim, Doa Kim, Hyunwoo Song, Kwang Suk Lim, Sehyun Chae

**Affiliations:** 1 Department of Biotechnology and Bioengineering, College of Art, Culture and Engineering, Kangwon National University, Chuncheon 24341, Republic of Korea; 2 Department of Smart Health Science and Technology, Kangwon National University, Chuncheon 24341, Republic of Korea; 3 Institute of Fermentation and Brewing, Kangwon National University, Chuncheon 24341, Republic of Korea

**Keywords:** spatial transcriptomics, next-generation sequencing, in situ hybridization, brain aging, neurodegenerative disease, glioblastoma

## Abstract

The brain is a spatially organized tissue where the molecular characteristics of each cell are closely linked to its anatomical location. However, conventional bulk and single-cell RNA sequencing lose this spatial context during the tissue separation process. Spatial transcriptomics (ST) overcomes these limitations by measuring gene expression while preserving the positional information of cells within intact tissues, making it a powerful approach for elucidating the cellular and molecular heterogeneity that defines brain structure and disease. This review summarizes the two main types of ST technology: next-generation sequencing (NGS)-based platforms (Visium, Stereo-Seq, Slide-Seq) and in situ platforms (MERFISH, seqFISH+, Xenium). NGS-based platforms provide unbiased whole-transcriptome profiling across extensive tissue regions, while in situ platforms offer subcellular resolution within individual cells. We aim to assist in platform selection by comparing the principles, advantages, and limitations of each platform. Next, we focus on how spatial sequencing (ST) has been utilized to analyze the spatial heterogeneity of aging and diseased brains, and examine region- and cell-type changes observed in brain aging, the lesion-related microenvironments of Alzheimer’s and Parkinson’s diseases, and the spatially isolated tumor cell states and immunosuppressive environments of glioblastoma. We also introduce the key brain ST data resources that underpin these studies. Collectively, ST is emerging as an essential tool for understanding the spatial logic of brain function and pathology, demonstrating increasingly greater potential in the field of precision medicine.

## 1. Introduction

The brain is one of the most spatially organized tissues in the human body, and the molecular identity and function of each cell are closely linked to its location within laminar, domain, and microenvironmental structures. Therefore, to understand how the brain functions and how it becomes dysfunctional in disease, gene expression must be measured in a way that preserves this spatial organization. However, conventional bulk RNA sequencing reports only the average transcription of all cells in a sample, obscuring the cellular diversity and spatial structure that underlie brain function [1]. This limitation is consequential for brain disorders, where pathology is frequently focal and where the identity of the affected cells and their location relative to a lesion carry both mechanistic and diagnostic meaning.

Single-cell RNA sequencing (scRNA-Seq) has significantly advanced this field by analyzing transcriptional heterogeneity at the individual-cell level. However, because scRNA-Seq requires tissue dissociation, the unique spatial coordinates of each cell are permanently lost. This spatial information is not incidental: the layered arrangement of cortical neurons, the proximity of immune cells within lesions, and the local niches of tumors are all spatially defined features that decisively shape the physiology and pathology of the brain [2]. Moreover, position itself encodes molecular regulation: morphogen gradients pattern the cortical layers, and locally restricted ligand–receptor signaling within tissue niches continuously shapes the transcriptional state of resident cells, so that where a transcript is expressed is inseparable from how its expression is controlled. Spatial transcriptomics (ST) was developed out of the need to analyze gene expression while maintaining positional context, and it is recognized as a groundbreaking methodological advance in tissue biology [3].

ST technologies are broadly divided into two complementary categories. NGS-based methods enable unbiased whole-transcriptome analysis across relatively wide tissue regions by using spatially barcoded substrates to capture transcripts and reconstruct their locations after sequencing [4]. In contrast, in situ-based methods achieve single-cell to subcellular resolution for predefined gene panels by directly detecting transcripts within tissues through sequential fluorescence hybridization or in situ sequencing [5]. The two approaches differ in spatial resolution, transcriptomic scope, and experimental complexity, and are increasingly used in combination depending on the research question.

Several reviews have investigated the principles of ST technology [6,7] and covered applications across neuroscience and cancer [8]. However, the brain presents unique yet common challenges: brain function and pathology are primarily governed by spatially organized heterogeneity, and specific cell populations and anatomical regions exhibit selective vulnerability and locally distinct molecular states [9]. This review focuses on how ST is being used to analyze this spatial heterogeneity in the aging and diseased brain. First, we compare representative NGS-based (Visium, Stereo-Seq, Slide-Seq) and in situ-based (MERFISH, seqFISH+, Xenium) platforms and highlight practical considerations that guide platform selection. We then examine ST applications in three contexts, demonstrating spatial heterogeneity in brain aging, neurodegenerative disease, and glioblastoma, and briefly introduce key brain ST data resources supporting these studies. By organizing recent findings around molecularly defined cell states and the niches they form, we aim to provide a synthesis that connects technological capability to mechanistic, and ultimately, clinical questions in brain aging and disease.

This article is a narrative review. Relevant literature was identified through searches of PubMed, Web of Science, and Google Scholar covering publications up to June 2026, using combinations of the terms “spatial transcriptomics”, “brain aging”, “Alzheimer’s disease”, “Parkinson’s disease”, and “glioblastoma”, together with the names of individual platforms (e.g., Visium, Stereo-Seq, Slide-Seq, MERFISH, seqFISH+, Xenium). We prioritized peer-reviewed primary studies that applied spatial transcriptomics to brain tissue, together with foundational methodological and review articles; non-spatial studies were cited selectively to provide mechanistic or clinical context. Because this is a focused, non-systematic synthesis rather than a systematic review, the cited literature is intended to be representative rather than exhaustive.

## 2. Spatial Transcriptomics Technologies

### 2.1. Overview and Classification

**Figure 1:**
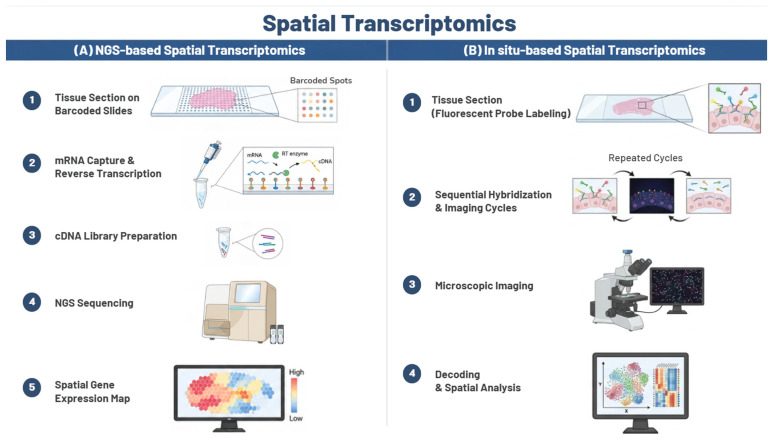
Overview of NGS-based and in situ-based spatial transcriptomics workflows. (**A**) Workflow of the NGS-based approach, including spatial barcoding, mRNA capture, reverse transcription, library preparation, sequencing, and reconstruction of the spatial gene-expression map. (**B**) Workflow of the in situ-based approach, featuring sequential hybridization and imaging cycles, microscopic imaging, and signal decoding for high-resolution spatial profiling. NGS, next-generation sequencing; mRNA, messenger RNA; cDNA, complementary DNA.

Spatial transcriptomics measures gene expression directly within an intact tissue section, so that each transcriptional measurement retains its location in the original tissue. Current platforms fall into two broad categories that differ in how positional information is obtained (Figure 1). NGS-based methods encode location through spatially barcoded capture substrates and read out transcripts by sequencing, providing largely unbiased, transcriptome-wide coverage over comparatively large tissue areas [6,10]. In situ-based methods instead identify transcripts in place through repeated rounds of fluorescence hybridization or in situ sequencing, yielding very high spatial resolution and single-molecule sensitivity for a targeted set of genes [7]. In practice, the two families trade off along three axes—spatial resolution, transcriptomic breadth, and experimental and computational complexity—so that the most suitable platform depends on the biological question, the tissue type, and the scale of the study. Historically, the field progressed from low-resolution, array-based capture toward both subcellular sequencing and single-molecule imaging, and this trajectory continues to narrow the trade-off between resolution and transcriptomic breadth. The brain has been a recurring proving ground for these methods, because its highly stereotyped yet locally heterogeneous architecture both demands and rewards spatial resolution. The representative platforms discussed below are compared in Table 1.

### 2.2. NGS-Based Platforms

Visium (10x Genomics) is the most widely adopted NGS-based platform and has effectively standardized spatial barcoding [11]. Tissue is placed on a glass slide arrayed with spatially barcoded reverse-transcription primers; after permeabilization, released mRNAs are captured on poly(dT) primers, and reverse transcription incorporates a spatial barcode and a unique molecular identifier into the cDNA, which is then sequenced and mapped back to tissue coordinates [11,20]. Its principal strengths are unbiased whole-transcriptome coverage requiring no predefined gene panel and the ability to survey large tissue areas, making it well-suited to discovery-oriented studies [6,7,21]. Because capture is spot-based, however, its resolution (~55 µm) does not reach the single-cell level, and each spot may contain several cells [11]; lateral diffusion of mRNA during capture can also blur the spatial signal [22]. Its accessibility and applicability to both fresh-frozen (FF) and formalin-fixed paraffin-embedded (FFPE) tissue have established it as a standard tool across tumor, brain, and developmental studies [12,23]. The recent Visium HD assay further increases resolution to a continuous grid of 2-µm bins, narrowing the gap toward single-cell measurements while retaining whole-transcriptome capture, although assigning the binned signal to individual cells still depends on downstream computational deconvolution [12].

Stereo-Seq achieves substantially higher resolution using densely patterned DNA nanoballs (DNBs) as capture units [13]. Each DNB carries a coordinate identity; after a tissue section is placed on the chip and permeabilized, captured transcripts are reverse-transcribed and sequenced to reconstruct expression at subcellular (~220 nm) resolution over large fields of view [13]. Its capacity to resolve fine structures has made it particularly valuable for mapping the cellular organization of complex tissues, including the developing and adult brain [13]. This combination of subcellular resolution, broad coverage, and whole-transcriptome readout is particularly powerful for discovery in development and neural tissue, and the introduction of Stereo-Seq V2 has extended its use from FF to FFPE samples [14]. The main cost is practical: the very high resolution generates large, complex datasets with correspondingly heavy computational demands [14,22].

Slide-Seq captures transcripts on DNA-barcoded microbeads (~10 µm) arrayed on a slide, whose positions are decoded in advance by sequencing [4]. Released mRNAs bind the beads, and subsequent reverse transcription and sequencing reconstruct expression at near-single-cell resolution while retaining whole-transcriptome coverage [4]. Slide-Seq V2 improved library preparation and bead packing to markedly increase the RNA capture efficiency and sensitivity over the original version [15]. The need to decode bead positions beforehand and the relatively modest capture efficiency of the first-generation method were its main limitations [4,15], but the platform is now widely used where near-cellular resolution is required across diverse tissues [24].

### 2.3. In Situ-Based Platforms

MERFISH (multiplexed error-robust fluorescence in situ hybridization) detects individual RNA molecules in place at single-molecule sensitivity [5]. Each RNA species is assigned a binary barcode that is read out over successive rounds of hybridization and imaging using encoding and readout probes; the presence or absence of signal in each round defines the barcode, allowing thousands of transcripts to be identified and quantified together with their positions [5,16]. A defining feature is its error-robust barcoding scheme, which corrects detection errors and improves data reliability [16], making the method especially valuable for cell-type classification and analyses of intercellular organization [25,26]. Its limitations arise from the repeated hybridization–imaging cycles, which are time-consuming and technically demanding and can suffer from optical crowding at high transcript density [27]; as with all targeted methods, detectable genes are constrained by a predesigned probe panel rather than being fully unbiased [28]. To offset this constraint, targeted measurements are increasingly paired with computational imputation that transfers information from matched single-cell references, extending the effective gene coverage toward the transcriptome [28].

seqFISH+ extends sequential FISH [29] by using combinatorial barcoding to profile up to ~10,000 genes at single-cell to subcellular resolution [17]. Transcripts are encoded by combinatorial barcodes decoded across multiple imaging cycles, with readout probes stripped and replaced between rounds [17,28]; by imaging only a fraction of the transcriptome per cycle, the method effectively mitigates optical crowding while preserving high multiplexing and sensitivity [17]. These properties make seqFISH+ well-suited to distinguishing closely related cell types and resolving tissue architecture, although the extended multi-cycle workflow and the need for specialized imaging infrastructure remain practical constraints [17,30].

Xenium (10x Genomics) combines target-specific probes with signal amplification and iterative fluorescence imaging to localize transcripts at single-cell to subcellular resolution [18]. It supports both FFPE and FF tissue, giving it strong applicability to clinical and pathology samples, and can profile up to several thousand genes [18,31]. As with the other imaging-based methods, its gene content is limited by a predesigned panel, and the high-resolution imaging and repeated hybridization steps entail relatively high instrument and analysis costs [18,19]. Xenium is increasingly applied in tumor-microenvironment, neural, and clinical studies where precise, cell-resolved spatial expression is required [19]. Because it bridges high-resolution imaging with routine clinical specimen formats, Xenium has been positioned as one of the more translation-ready imaging platforms, and its expanding gene panels are progressively reducing the historical gap between targeted and unbiased approaches [18,19].

Together, these platforms illustrate the complementary nature of the two approaches: NGS-based methods favor unbiased, transcriptome-wide discovery across large areas, whereas in situ-based methods deliver targeted, high-resolution, single-cell measurements (Table 1). This complementarity directly informs platform selection for brain studies, as discussed below.

The platforms compared above were selected because they are among the most widely adopted and best-benchmarked representatives of the two methodological families, and together they span the practical range of spatial resolution, transcriptomic breadth, and tissue compatibility most relevant to brain research. They are not an exhaustive catalogue. Several other spatial platforms are in active use, including the imaging- and probe-based CosMx Spatial Molecular Imager, which profiles RNA and proteins at single-cell to subcellular resolution [32], and the region-based GeoMx Digital Spatial Profiler, which quantifies proteins and RNA within user-defined regions of interest [33], as well as additional approaches such as STARmap, HybISS, and DBiT-Seq. These methods were deliberately not added to Table 1 in order to keep the comparison focused on platforms most frequently applied to brain tissue; conceptually, however, they occupy the same trade-off space defined by resolution, throughput, and multiplexing, and the selection principles outlined here also apply to them.

### 2.4. Computational Analysis of Spatial Transcriptomic Data

The biological value of spatial transcriptomic data depends heavily on computational analysis, because the raw measurements rarely map directly onto single cells or onto molecularly defined tissue structures. Spot-based NGS methods typically capture mixtures of several cells per spot, whereas imaging-based methods resolve single cells but only for a predesigned panel of genes; bridging these gaps is the task of a rapidly growing analytical toolkit. The first major step is cell-type deconvolution or mapping, in which single-cell RNA-Seq references are used to estimate the cellular composition of each spatial measurement. Probabilistic and regression-based methods such as cell2location [34] and robust cell-type decomposition (RCTD) [35], together with approaches such as SPOTlight [36] and Tangram [37], project molecularly defined cell identities onto spatial coordinates, allowing the spatial distribution of specific cell types to be recovered even from multicellular spots. The reliability of every downstream biological conclusion therefore depends on the appropriateness of these analytical choices, which has made methodological rigor a central concern in the field.

A second step is the identification of spatial domains, contiguous regions that share a coherent molecular profile. Methods such as BayesSpace [38] and SpaGCN [39] integrate gene expression with spatial neighborhood, and in some cases tissue histology, to delineate tissue architecture in a data-driven manner. A third and increasingly important step is the inference of cell–cell communication: tools such as CellChat [40] and CellPhoneDB [41] use ligand–receptor co-expression to reconstruct signaling between neighboring cell populations, an analysis that is particularly informative when paired with spatial information because it links physical proximity to molecular crosstalk. In the disease contexts discussed below, these analyses are what convert spatial maps into mechanistic insight defining the disease-associated niches of the aging and degenerating brain and the immunosuppressive signaling circuits of the glioblastoma microenvironment. Because benchmarking studies show that the performance of these methods varies with platform, resolution, and tissue type, careful method selection and validation against orthogonal data remain essential [19,31].

Beyond these core tasks, spatial data are routinely integrated with comprehensive single-cell atlases to transfer fine-grained cell-type labels, and with neighborhood- or niche-analysis methods that group recurrent local cellular compositions into higher-order tissue units. Trajectory and spatially aware modeling can then relate these units to gradients of differentiation or disease progression across the tissue. For the brain, where the cytoarchitecture is highly ordered, such analyses are essential for translating raw spatial measurements into interpretable maps of how molecularly defined cell types are arranged, how they communicate, and how these relationships are remodeled in aging and disease. The mechanistic findings summarized in the following sections rest directly on this analytical foundation.

A particularly active and rapidly evolving direction is the joint analysis of spatial transcriptomic data with the matched histology images and additional omics layers that increasingly accompany them. Because most sequencing-based platforms (e.g., Visium and Stereo-Seq) acquire a hematoxylin-and-eosin (H&E) or comparable histological image of the same section, tissue morphology and the transcriptome describe the same physical coordinates and can be modeled jointly. Deep-learning methods exploit this correspondence in several complementary ways: histology-aware graph and self-supervised models (e.g., stLearn, SpaGCN, and DeepST) use morphological features to refine spatial-domain identification and to denoise or enhance gene-expression estimates; image-to-expression models predict spatially resolved transcriptomes directly from H&E images, offering a route to extend or super-resolve sparse spatial measurements; and emerging histology–transcriptomics foundation models learn shared representations across the two modalities. More broadly, advanced artificial-intelligence frameworks now integrate spatial transcriptomics with H&E imaging and multidimensional omics (proteomic, epigenomic, and metabolomic) data through attention-, graph-, and contrastive-learning-based multimodal fusion, yielding a more complete molecular and morphological picture of the tissue than any single modality alone [42,43]. For the brain, whose laminar and regional cytoarchitecture is well-captured by routine histology, such image-aware and multimodal approaches are especially promising for linking morphological landmarks to molecular niches, but their practical adoption will nonetheless depend on careful cross-platform validation, model interpretability, and data standardization.

## 3. Spatial Transcriptomics of the Aging and Diseased Brain

Across aging, neurodegeneration, and malignancy, the brain does not change uniformly; rather, particular cell populations, anatomical regions, and local microenvironments are affected selectively. Spatial transcriptomics is therefore especially suited to these settings because it links transcriptional change to its precise location in the tissue. Here, we summarize how ST has been used to resolve spatial heterogeneity in three representative contexts brain aging, neurodegenerative disease, and glioblastoma (Table 2). In addition to this application-level summary, we further listed representative molecular characteristic marker genes and cell state programs identified across these conditions through spatial studies (Table 3). We focus on these three because they span the major modes of brain pathology gradual physiological decline, progressive neuronal loss, and uncontrolled proliferation and because each has been studied with spatial methods in sufficient depth to illustrate distinct lessons about how molecular heterogeneity is organized in space. Throughout this section, we distinguish findings obtained from human tissue from those derived in animal models, because the balance of evidence differs markedly across conditions: glioblastoma, and to a large extent, Alzheimer’s disease, have been profiled directly in human specimens, whereas much of the brain-aging evidence rests on mouse models and the substantia nigra findings in Parkinson’s disease are still inferred largely from non-spatial and review-level studies. Observations from model systems should therefore be extrapolated to human brain pathology with appropriate caution.

### 3.1. Brain Aging

Brain aging is increasingly understood not as a uniform, global decline but as a process of molecular and structural remodeling that occurs selectively in specific cell populations and tissue microenvironments [9]. Because this heterogeneity is spatially organized, ST, which couples gene-expression changes to positional information, has become an effective approach for characterizing it [11]. Spatial analyses indicate that age-associated molecular changes are concentrated in specific brain regions. In particular, the subcortical white matter shows increased expression of inflammation- and immune-related genes, marking it as a major spatial hotspot of change during aging [44,45]. These spatial findings were obtained primarily in the aging mouse brain [46], and directly comparable spatial transcriptomic datasets from human brain aging remain limited at present. At the cellular level, these alterations are more pronounced in glial cells, astrocytes, microglia, and oligodendrocytes than in neurons, indicating that brain aging is closely linked to a non-neuronal, glia-centered functional reorganization rather than simple neuronal loss [44,45]. At the molecular level, a substantial fraction of aging astrocytes adopts an A1-like reactive state induced by microglia-derived signals and marked by the upregulation of complement components such as *C3* and *Serping1*, which is associated with the loss of homeostatic support and potential neurotoxicity [60]. Aged microglia, in turn, shift toward activated states linked to the clearance of degraded myelin, a metabolic burden that accumulates in white-matter tracts and helps explain why these regions emerge as spatial hotspots of inflammation [44,60]. Intercellular interactions are likewise spatially regulated: the activation of astrocytes and microglia during aging is shaped by their interactions with vascular cells and neighboring glia, and white-matter regions such as the corpus callosum show a particularly concentrated accumulation of glia and immune activity [44,46]. Age-related changes in the neurovascular unit, including altered astrocyte–endothelial signaling at the blood–brain barrier, are also spatially concentrated and contribute to the regional inflammatory profile [46]. Together, these findings show that brain aging is a spatially organized process centered on specific cell populations and tissue structures, and that ST is a valuable tool for dissecting its molecular mechanisms and defining region-selective vulnerability. They also reframe brain aging as an active, spatially patterned remodeling of glial and vascular compartments rather than a passive, uniform decline, with direct implications for how age-related risk for neurodegeneration is distributed across the tissue.

### 3.2. Neurodegenerative Disease

Neurodegenerative diseases are characterized by the selective vulnerability of particular neuronal populations and by lesion-centered pathological change [9]. Because their pathophysiology varies with both cell type and tissue location, ST has become an important tool for resolving this spatial heterogeneity at high precision [11]. In Alzheimer’s disease (AD), the regions surrounding amyloid plaques show increased expression of inflammatory and immune-related genes, forming a lesion-centered microenvironment, the plaque-associated niche [47]. Within this niche, astrocyte- and microglia-centered responses are spatially concentrated, and cells adjacent to the lesion adopt a shared pathological transcriptional state; the gene-expression differences between the lesion core and the surrounding tissue are closely tied to disease onset and spread [47,48]. At the molecular level, plaque-proximal microglia converge on a conserved disease-associated microglia (DAM) program, characterized by the downregulation of homeostatic genes and induction of markers such as *Trem2*, *Apoe*, *Cst7*, and *Itgax*, while neighboring astrocytes upregulate reactive markers; spatial profiling shows that these molecular states are organized in concentric layers around the plaque core [61]. The existence of such graded molecular states, rather than a binary affected-versus-unaffected distinction, is itself a key insight that only spatially resolved measurement can provide. Parkinson’s disease (PD) exhibits an analogous spatial logic. The selective vulnerability of dopaminergic neurons in the substantia nigra is pronounced, with specific neuronal populations lost preferentially within spatially restricted regions [49]. At the molecular level, this selective vulnerability has been linked to a high basal metabolic and oxidative burden and to *α-synuclein* (SNCA) pathology, and spatial approaches are beginning to map how these cell-intrinsic programs intersect with locally restricted neuroinflammatory signaling from neighboring glia [50]. In both diseases, astrocyte and microglial activation increases selectively around specific lesions and is closely associated with neuronal loss [47,50]. Comparative spatial studies further suggest that although the triggering pathology differs, AD and PD share recurring motifs of lesion-centered glial activation, pointing to common spatially organized mechanisms of neurodegeneration that ST is well-placed to define. These observations indicate that neurodegenerative disease is not a single, diffuse pathological process but one governed by spatial microenvironments organized around specific lesions, and ST thus provides an important basis for understanding disease progression and designing lesion-specific therapeutic strategies.

### 3.3. Glioblastoma

Glioblastoma (GBM), the most aggressive primary brain tumor in adults, is defined by pronounced cellular heterogeneity and a complex tumor microenvironment [51,62]. Because this heterogeneity is confined to particular tissue regions and cell populations, ST, which preserves positional information while profiling gene expression, has become a central analytical tool [51]. Spatial studies show that GBM tumor cells occupy four principal cellular states—neural-progenitor-like (NPC-like), oligodendrocyte-progenitor-like (OPC-like), astrocyte-like (AC-like), and mesenchymal-like (MES-like) and that these states are spatially segregated [51,52]. Each state is defined by characteristic molecular markers—neural-progenitor-like (e.g., *SOX2*, *DLL3*), oligodendrocyte-progenitor-like (e.g., *OLIG1*, *PDGFRA*), astrocyte-like (e.g., *GFAP*, *AQP4*), and mesenchymal-like (e.g., CD44, *VIM*) and the mesenchymal-like program is driven in part by hypoxia and nuclear factor κB (NF-κB) signaling concentrated within the perinecrotic niche [52,54]. Oligodendrocyte-progenitor-like cells are relatively enriched in the tumor core, whereas radial-glial stem-like cells are concentrated within the invasive neural niche [53]; the expression differences between the tumor core and surrounding tissue offer important clues to the routes of tumor formation and spread [51,53]. The spatial organization of the tumor microenvironment is an equally important feature. The GBM microenvironment can be divided into two functionally distinct compartments, the perinecrotic and perivascular niches [54]. In the perinecrotic niche, immunosuppressive transcriptional states of tumor cells and the functional impairment of immune cells are spatially concentrated, and the hypoxic environment reinforces this immunosuppressive structure; hypoxic tumor cells drive the immunosuppressive phenotypic conversion of tumor-associated macrophages (TAMs) through CCL8 and IL1B signaling [54]. In the perivascular niche, in contrast, tumor cells with stem-like properties are maintained through close association with the vasculature, and the surrounding signaling environment supports their self-renewal and resistance to treatment; the spatial separation of this niche from the hypoxic core helps explain how distinct functional programs coexist within a single tumor [54]. Cell–cell interactions also vary with location: microglia are distributed mainly at the tumor margin, where they participate in invasion and local immune regulation, whereas monocyte-derived macrophages accumulate in the tumor core and perivascular regions and contribute to the immunosuppressive microenvironment [55,56]. Finally, the molecular features of the surgical resection margin are closely linked to recurrence, and margin-specific genes including a shared glioma-infiltrative signature and epidermal growth factor receptor (EGFR) promote tumor-cell migration and invasion [57].

Collectively, these results portray GBM as a spatially organized structure in which diverse cell states and functions are arranged across the tissue, and they position ST as a key approach for interpreting the spatial architecture of the tumor microenvironment and designing tailored therapeutic strategies. Importantly, because these programs are defined as much by their location as by their molecular content, therapeutic strategies that ignore spatial organization risk addressing only part of the tumor.

### 3.4. Multiple Sclerosis

Multiple sclerosis (MS) is the most common inflammatory demyelinating disease of the central nervous system and is characterized by spatially heterogeneous lesions whose cellular composition shifts as they evolve from active to chronic stages. Because lesion identity is defined by the spatial arrangement of demyelination, immune-cell infiltration, and glial reactivity features that are lost when tissue is dissociated, MS is particularly well-suited to spatially resolved analysis. Using single-cell-resolution in situ sequencing in post-mortem human MS tissue together with a mouse experimental autoimmune encephalomyelitis (EAE) model, one study mapped the cellular architecture of evolving lesions and identified disease-associated glial and immune states concentrated at the lesion rim, supporting a centrifugal model of active lesion expansion [58]. Complementary spatial transcriptomic profiling of human white- and grey-matter lesions resolved a distinct gene-expression signature surrounding active lesions and reconstructed trajectories predicting how normal-appearing white matter progresses toward active and mixed active–inactive lesions [59]. Together, these studies show that spatial approaches can distinguish lesion stages and localize the cell-type-specific programs that drive lesion progression. As for the other diseases discussed here, however, much of this mechanistic detail derives from a small number of post-mortem cohorts and animal models and will require validation in larger human datasets.

## 4. Brain Spatial Transcriptomics Data Resources and Current Challenges

The growing volume of brain ST data is supported by several major public resources. Because spatial datasets are large, heterogeneous, and methodologically diverse, shared reference resources are essential for comparing results across studies and for grounding new data in established molecular cell-type definitions. The 10x Genomics platform generates and distributes Visium-based data and provides dedicated analysis and visualization tools (Space Ranger, Loupe Browser); its widely used human dorsolateral prefrontal cortex (DLPFC) dataset has become a community reference, and the recent Visium HD technology pushes the resolution to ~2 µm, approaching single-cell scale [63,64]. The Allen Brain Cell Atlas (ABC Atlas) integrates large-scale single-cell and spatial data, combining roughly seven million scRNA-Seq profiles with ~4.3 million MERFISH measurements (using 500~1100-gene panels) to define a whole-mouse-brain atlas of more than 5000 transcriptomic clusters; it is complemented by human middle temporal gyrus data, alignment to the Common Coordinate Framework (CCF v3), and a 2024 imputation dataset (~8000 genes) that extends panel-limited spatial data toward the transcriptome scale [65,66,67,68]. The Single Cell Portal (SCP), operated by the Broad Institute, is a community-driven, open-access platform where users can upload, share, and interactively explore single-cell and spatial data without coding; its Slide-Seq/Slide-Seq V2 datasets (~10 µm resolution) underpin a three-dimensional spatial atlas of the whole mouse brain and are linked to the BRAIN Initiative Cell Census Network (BICCN) [69,70]. Together, these resources provide integrated, cross-species reference maps and analytical environments that serve as essential infrastructure for brain ST research. Cross-species correspondence between mouse atlases and human reference datasets is especially valuable for the brain, allowing molecular cell types defined in model systems to be related to their human counterparts.

Despite this progress, several challenges temper the interpretation of brain ST data. Spot-based NGS methods often capture multiple cells per measurement, whereas imaging-based methods are restricted to predesigned gene panels, so neither resolution nor transcriptomic breadth is simultaneously complete. Systematic benchmarking has further shown that sensitivity, specificity, and cell-segmentation accuracy vary considerably across imaging platforms and between FFPE and fresh-frozen tissue, underscoring the need for careful platform choice and standardized analysis [19,31]. Practical platform selection therefore depends on matching resolution, transcriptomic coverage, tissue compatibility, and cost to the biological question [22]. Differences in tissue handling, probe-panel design, and segmentation pipelines also introduce batch effects that complicate cross-study integration, and computational imputation, though valuable for extending panel-limited data toward the transcriptome scale, can introduce its own biases that should be validated against orthogonal measurements [68]. For the brain in particular, dense neuropil, fine regional architecture, and the requirement for accurate single-cell assignment make reproducibility and standardization especially important as datasets are integrated across studies and resources. Beyond two-dimensional sections, the assembly of serial sections into three-dimensional reconstructions is increasingly used to capture how molecular domains are organized through the volume of a structure rather than within a single plane, which further increases the demand for standardized acquisition and analysis.

A further set of limitations is biological and clinical rather than purely technical, and these temper how confidently current findings can be generalized. Many brain ST studies are based on small numbers of donors with limited demographic and genetic diversity, so inter-individual variability differences in age, sex, comorbidity, post-mortem interval, and genetic background are often underexplored and may confound the spatial signatures reported. Human studies depend heavily on post-mortem or surgically resected tissue, in which RNA integrity, agonal state, and FFPE-related degradation can bias measured expression, whereas a substantial share of mechanistic insight still derives from animal models whose correspondence to human disease is incomplete. Several findings synthesized above rest on single studies, or in the case of Parkinson’s disease, on extrapolation from non-spatial work, and a few rely on preprints that have not yet completed peer review; such results should be regarded as provisional until independently replicated. Translating ST-derived spatial signatures into diagnostic or therapeutic tools faces additional barriers, including the absence of standardized and clinically validated assays and reference ranges, high cost and limited throughput, the need for reproducible cell-segmentation and analysis pipelines, and the current scarcity of real-world clinical workflows. Recognizing these biological and clinical constraints is essential both for reading the existing literature critically and for setting realistic expectations for clinical translation.

## 5. Conclusions

In this review, we compared the principles and characteristics of NGS-based and in situ-based ST platforms and examined how they have been applied to resolve spatial heterogeneity in the aging and diseased brain. NGS-based methods (Visium, Stereo-Seq, Slide-Seq) offer comprehensive, transcriptome-wide profiling over large tissue areas, but except for subcellular approaches such as Stereo-Seq, often fall short of single-cell resolution; in situ-based methods (MERFISH, seqFISH+, Xenium) provide single-molecule or single-cell resolution and high multiplexing but are constrained by experimental complexity and predesigned gene panels. The two families are thus complementary, and appropriate platform selection according to the biological question and tissue type is essential.

Applied to the brain, ST has revealed region-selective molecular changes in aging, lesion-specific microenvironments in Alzheimer’s and Parkinson’s diseases, the spatially segregated tumor-cell states and immunosuppressive niches of glioblastoma, and intercellular interactions that are regulated by spatial context. Large-scale resources such as the Allen Brain Cell Atlas, 10x Genomics, and the Single Cell Portal integrate brain data across species and platforms and support three-dimensional atlases and imputation-based transcriptome expansion, functioning as critical data infrastructure for the field. Looking forward, ST is expected to advance through improved single-cell resolution, transcriptome-wide in situ profiling, integration with multi-omics data, and the development of computational methods for efficiently handling large spatial datasets; concrete examples already include the extension of Stereo-Seq V2 to FFPE tissue, the introduction of Visium HD, and the continued expansion of Xenium gene panels [14,22]. By enabling a more precise understanding of brain pathophysiology and supporting the development of spatially informed, personalized therapeutic strategies, these advances are poised to drive innovation across neuroscience and precision medicine.

Two directions are especially promising. First, ST is increasingly integrated with other spatially resolved modalities including proteomics, chromatin accessibility, and metabolomics so that transcriptional states can be interpreted alongside the regulatory and functional layers that accompany them; recent spatial multi-omics analyses of the glioma resection margin illustrate how such integration can define clinically meaningful molecular signatures [57]. Such integration is increasingly pursued across modalities and disease settings: spatial proteomic and antibody-based imaging methods (e.g., CODEX [co-detection by indexing] and protein-capable in situ panels) [71] resolve the protein correlates of transcriptional states; spatial epigenomic assays such as spatial ATAC-Seq [assay for transposase-accessible chromatin using sequencing] map chromatin accessibility onto tissue coordinates [72]; and mass-spectrometry imaging adds a spatially resolved metabolomic layer [73]. In glioblastoma, multi-omic profiling of the resection margin links transcriptional infiltrative programs to clinically relevant tissue features [57]; in Alzheimer’s disease, pairing spatial transcriptomics with proteomic mapping of amyloid and tau deposition can connect molecular niches to the underlying pathology [74]; and in brain aging, joint transcriptomic–epigenomic readouts help distinguish the regulatory drivers of glial activation from its downstream consequences [72]. Because no single modality simultaneously captures regulation, transcription, protein, and metabolism, spatially resolved multi-omics is likely to become central to mechanistic and translational brain studies. Second, by anchoring molecular states to defined tissue locations, ST provides a route toward spatially informed precision medicine, in which region- and niche-specific signatures such as the infiltrative margin programs of glioblastoma or the lesion-associated niches of neurodegeneration could inform diagnosis, prognosis, and the selection of spatially targeted therapies. Importantly, the route to clinical application differs across these diseases. In glioblastoma, where surgical resection and biopsy are part of routine care, spatially resolved signatures obtained directly from patient tissue could in principle be incorporated into diagnostic and therapeutic decision-making. In Alzheimer’s and Parkinson’s disease, in contrast, brain biopsy is not an acceptable diagnostic procedure in living patients; the contribution of ST is therefore necessarily more indirect, lying in mechanistic insight and biomarker discovery from post-mortem or model tissue that may in turn inform fluid- or imaging-based biomarkers rather than direct spatial assays in patients. It should be emphasized, however, that these clinical applications remain largely aspirational: at present, the overwhelming majority of brain ST studies are confined to basic and translational research, very few validated clinical workflows exist, and substantial hurdles in standardization, cost, turnaround time, regulatory approval, and prospective validation must be overcome before spatial signatures can enter routine diagnostic or therapeutic practice. Realizing this potential will require standardized and clinically robust workflows, but it positions ST as a bridge between molecular neuroscience and translational application. Together with community data resources and standardized analytical pipelines, these developments should make spatially resolved molecular information progressively more accessible for both basic and clinical neuroscience.

## Figures and Tables

**Table 1:** Comparison of NGS-based and in situ-based spatial transcriptomics platforms.

Platform	Approach	Spatial Resolution	Transcriptomic Coverage	Tissue	Throughput/Multiplexing	Relative Complexity & Cost	Representative Applications
Visium [11,12]	NGS-based	~55 µm (spot)	Whole transcriptome (unbiased)	FF, FFPE	n/a (genome-wide)	Low–moderate; standardized	Tumor, brain, developmental biology
Stereo-Seq [13,14]	NGS-based	~220 nm (subcellular)	Whole transcriptome	FF, FFPE (V2)	n/a (genome-wide)	High (large data, heavy compute)	Development, neural tissue, cell–cell interaction
Slide-Seq (V2) [4,15]	NGS-based	~10 µm (near single-cell)	Whole transcriptome	FF	n/a (genome-wide)	Moderate (bead decoding)	Near-cellular profiling across tissues
MERFISH [5,16]	In situ	~100 nm (single-molecule)	Targeted (100s–~10,000 genes)	FF, FFPE	High	High (multi-cycle imaging)	Cell-type mapping, intercellular organization
seqFISH+ [17]	In situ	~100 nm (subcellular)	Targeted (up to ~10,000 genes)	FF	Very high	High (multi-cycle, specialized imaging)	Cell-type discrimination, tissue architecture
Xenium [18,19]	In situ	~200 nm (single-cell/subcellular)	Targeted (100s–~5000 genes)	FF, FFPE	High	Moderate–high; clinically oriented	Tumor microenvironment, neural, clinical

Abbreviations: FF, fresh-frozen; FFPE, formalin-fixed paraffin-embedded; NGS, next-generation sequencing; n/a, not applicable; V2, version 2.

**Table 2:** Representative applications of spatial transcriptomics in the aging and diseased brain.

Condition	Brain Region/Niche	Key	Primary Evidence (Model/Tissue)	Ref(s).
Brain aging	Subcortical white matter, corpus callosum	Region- and glia-selective inflammatory remodeling; white matter as an immune/inflammation hotspot; non-neuronal (astrocyte/microglia/oligodendrocyte) reorganization	Mouse brain (spatial); mouse mechanistic support	[44,45,46]
Alzheimer’s disease	Amyloid-plaque microenvironment (hippocampus, cortex)	Plaque-associated niche with spatially concentrated astrocyte/microglial inflammatory states; shared pathological transcriptional state near lesions	Human post-mortem brain (incl. preprint)	[47,48]
Parkinson’s disease	Substantia nigra	Spatially restricted, selective loss of dopaminergic neurons shaped by the local microenvironment	Review-based; direct human ST evidence limited	[49,50]
GBM tumor cell states	Tumor core vs. invasive neural niche	Four spatially segregated states (NPC-/OPC-/AC-/MES-like); OPC-like enriched in core, radial-glial stem-like in invasive niche	Human tumor tissue (spatial + scRNA-Seq)	[51,52,53]
GBM microenvironment	Perinecrotic & perivascular niches	Hypoxia-driven immunosuppression; TAM reprogramming via CCL8/IL1B; microglia at margin, monocyte-derived macrophages in core	Human tumor; supporting mouse models	[54,55,56]
GBM resection margin	Surgical resection margin	Margin-specific infiltrative signature including EGFR, linked to invasion and recurrence	Human tumor tissue (spatial multi-omics)	[57]
Multiple sclerosis	White-matter lesions; lesion rim	Stage-specific lesion architecture; disease-associated glia and immune states at the active-lesion rim; trajectories from normal-appearing white matter to active/mixed lesions	Human post-mortem brain + mouse EAE (spatial)	[58,59]

The “Primary evidence” column indicates the model system and tissue source underlying each finding. Evidence levels are not uniform across rows: several entries derive from single studies, animal models, or review-level synthesis rather than multiple independent human datasets, and model-based observations should be validated in human tissue before clinical extrapolation. Abbreviations: NPC-like, neural-progenitor-like; OPC-like, oligodendrocyte-progenitor-like; AC-like, astrocyte-like; MES-like, mesenchymal-like; GBM, glioblastoma; TAM, tumor-associated macrophage; EGFR, epidermal growth factor receptor.

**Table 3:** Representative molecular signatures resolved by spatial transcriptomics across the aging and diseased brain.

Condition	Cell Type/Niche	Representative Molecular Signature	Primary Evidence (Model/Tissue)	Ref(s).
Brain aging	A1-like reactive astrocytes	Complement induction (*C3*, *Serping1*); loss of homeostatic support	Mouse/in vitro	[45,60]
Brain aging	Aged microglia (white matter)	Activated, myelin-clearance-associated states	Mouse	[44,60]
Alzheimer’s disease	Disease-associated microglia (plaque niche)	*Trem2*, *Apoe*, *Cst7*, *Itgax*; loss of homeostatic genes	Mouse model (5xFAD); conserved in human	[61]
Parkinson’s disease	Vulnerable dopaminergic neurons (substantia nigra)	*α-synuclein* (SNCA) pathology; oxidative/metabolic stress programs	Review-based; human/model synthesis	[49,50]
Glioblastoma	Four tumor-cell states	*SOX2*/*DLL3* (NPC), *OLIG1*/*PDGFRA* (OPC), *GFAP/AQP4* (AC), CD44/*VIM* (MES)	Human tumor (scRNA-Seq–derived)	[52]
Glioblastoma	Perinecrotic niche/TAMs	Hypoxia and NF-κB signaling; CCL8, IL1B–driven TAM reprogramming	Human tumor; supporting mouse models	[54]
Glioblastoma	Resection margin	EGFR and a shared glioma-infiltrative signature	Human tumor tissue	[57]
Multiple sclerosis	Lesion-rim glia (active lesions)	Disease-associated microglia/astrocyte and immune-cell programs marking active-lesion rims; white-matter lesion-progression signatures	Human post-mortem brain + mouse EAE	[58,59]

As in Table 2, the “Primary evidence” column denotes the model system and tissue source. Signatures defined primarily in animal models (e.g., the disease-associated microglia program) or synthesized from review-level evidence (Parkinson’s disease) require further confirmation in human spatial datasets. Abbreviations: *C3*, complement component 3; SNCA, α-synuclein; NF-κB, nuclear factor κB; NPC, neural progenitor cell; OPC, oligodendrocyte progenitor cell; AC, astrocyte; MES, mesenchymal; TAM, tumor-associated macrophage; EGFR, epidermal growth factor receptor.

## Data Availability

No new data were created or analyzed in this study. Data sharing is not applicable to this article.

## Abbreviations

ST: spatial transcriptomics; NGS, next-generation sequencing; scRNA-seq, single-cell RNA sequencing; FF, fresh-frozen; FFPE, formalin-fixed paraffin-embedded; DNB, DNA nanoball; MERFISH, multiplexed error-robust fluorescence in situ hybridization; AD, Alzheimer’s disease; PD, Parkinson’s disease; GBM, glioblastoma; TAM, tumor-associated macrophage; EGFR, epidermal growth factor receptor; DLPFC, dorsolateral prefrontal cortex; ABC Atlas, Allen Brain Cell Atlas; SCP, Single Cell Portal; CCF, Common Coordinate Framework.
